# Gastric Alimetry in the Management of Chronic Gastroduodenal Disorders: Impact to Diagnosis and Health Care Utilization

**DOI:** 10.14309/ctg.0000000000000626

**Published:** 2023-08-17

**Authors:** Chris Varghese, Charlotte Daker, Alexandria Lim, Gabrielle Sebaratnam, William Xu, Bernard Kean, Chris Cederwall

**Affiliations:** 1 Department of Surgery, Auckland City Hospital, Auckland, New Zealand;; 2 Department of Gastroenterology, North Shore Hospital, Auckland, New Zealand;; 3 Wellington Regional Hospital, Wellington, New Zealand.

**Keywords:** gastroparesis, health economics, functional dyspepsia, body surface gastric mapping

## Abstract

**INTRODUCTION:**

Chronic gastroduodenal symptoms are frequently overlapping within existing diagnostic paradigms, and current diagnostic tests are insensitive to underlying pathophysiologies. Gastric Alimetry has emerged as a new diagnostic test of gastric neuromuscular function with time-of-test symptom profiling. This study aimed to assess the impact to diagnosis and health care utilization after the introduction of Gastric Alimetry into clinical care.

**METHODS:**

Consecutive data of patients from 2 tertiary centers with chronic gastroduodenal symptoms (Rome-IV defined or motility disorder) having integrated care and Gastric Alimetry testing were evaluated. Changes in diagnoses, interventions, and management were quantified. Pretest and posttest health care utilization was reported. A preliminary management framework was established through experiential learning.

**RESULTS:**

Fifty participants (45 women; median age 30 years; 18 with gastroparesis, 24 with chronic nausea and vomiting syndrome, and 6 with functional dyspepsia) underwent Gastric Alimetry testing. One-third of patients had a spectral abnormality (18% dysrhythmic/low amplitude). Of the remaining patients, 9 had symptoms correlating to gastric amplitude, while 19 had symptoms unrelated to gastric activity. Gastric Alimetry aided management decisions in 84%, including changes in invasive nutritional support in 9/50 cases (18%; predominantly de-escalation). Health care utilization was significantly lower post–Gastric Alimetry testing when compared with the average utilization cost in the year before Gastric Alimetry testing (mean ± SD $39,724 ± 63,566 vs $19,937 ± 35,895, *P* = 0.037).

**DISCUSSION:**

Gastric Alimetry aided diagnosis and management of patients with chronic gastroduodenal symptoms by enabling phenotype-informed care. The high majority of results aided management decisions, which was associated with reduced health care utilization.

## INTRODUCTION

Chronic gastroduodenal symptoms pose a diagnostic and therapeutic challenge. These symptoms are commonly seen in chronic gastroduodenal disorders, including functional dyspepsia (FD), chronic nausea and vomiting syndrome (CNVS), and in gastroparesis with delayed gastric emptying (1,2). However, recent evidence shows these disorders are clinically interchangeable because of overlapping symptom profiles and tests (3). More specific diagnostic pathways are therefore required to guide targeted care (4).

Gastric Alimetry (Alimetry, Auckland, New Zealand) is a new diagnostic device, combining noninvasive gastric electrical mapping with simultaneous symptom logging in a validated App (5,6). The gastric mapping data are capable of identifying patient subgroups with neuromuscular dysfunction, while the symptom data can help determine symptom origins (5,6).

Gastric Alimetry is now being introduced into practice, challenging existing models of care. As such, this study had 2 aims; first, to develop and refine an initial framework for the implementation of Gastric Alimetry in the routine management of gastroduodenal disorders and, second, to assess its influence on diagnosis and management in a consecutive series of 50 cases.

## METHODS

### Study design

A series of 50 consecutive patients with chronic gastroduodenal disorders investigated with Gastric Alimetry were retrospectively evaluated after ethical approval. Patients were recruited from 2 tertiary centers in the North Island of New Zealand receiving care from gastroenterologists with motility expertise (n = 2). Patients with a diagnosis of cannabinoid hyperemesis syndrome or cyclical vomiting syndrome were excluded.

All patients underwent standard Gastric Alimetry tests: 30-minute fasted test meal, followed by 4-hour postprandial recording with concurrent symptom logging (5,6). The standard test meal recommended by the manufacturer, for which validated spectral reference ranges are available, was used (approximately 480 kCal consisting of an oatmeal energy bar and nutrient drink) (7). Pretest and posttest test diagnoses were assessed, together with management changes implemented following Gastric Alimetry, to define clinical decision-making impact made on its basis. CNVS and FD were diagnosed per Rome-IV criteria (2), and gastroparesis was defined as a delayed gastric emptying test (GET) (8). Where gastric emptying data were available, all were scintigraphic studies. Gastroscopy and *Helicobacter pylori* testing were routine in both centers. Comorbidity data are summarized in the Supplementary Appendix (see Supplementary Digital Content 1, http://links.lww.com/CTG/A994).

The clinical impact of Gastric Alimetry was evaluated applying similar methodology and criteria previously used in a study addressing the impact of antroduodenal manometry (9). Charts were reviewed by an independent assessor, with a positive management impact defined as an outcome that established a new diagnosis or altered therapy (medication, endoscopic intervention, feeding) (9).

### Gastric Alimetry test phenotypes

A Gastric Alimetry phenotype set that is currently under iterative development was used (10). This classification approach was applied clinically, as based on recent Gastric Alimetry studies (7,11,12), the gastric physiology literature (10), and experience from applying these learnings in routine clinical practice, according to the following scheme:

Gastric Alimetry spectral analysis provides the following major phenotypes (10):

1. Dysrhythmic phenotype: Gastric Alimetry Rhythm Index <0.25, supported by low amplitudes <22 μV (13). Managed as per gastroparesis guidelines (1), regardless of GET status.
2. High sustained body mass index–adjusted amplitude: >70 μV (13). Consider distal obstruction and evaluate GET with consideration of pyloric therapies (13).
3. Normal spectral activity: Request GET if required (1). If delayed, prokinetics initiated and pyloric therapies considered (1); if normal, managed per symptom profile below.

Gastric Alimetry symptom profiling provides the following major phenotypes:

1. Normal spectral analysis and symptoms dependent on the gastric amplitude:
  - Sensorimotor: symptoms correlate with gastric amplitude, suggesting possible hypersensitivity/accommodation disorders. Consider therapy per postprandial distress syndrome (2).
  - Postgastric: symptoms trending upward late in postprandial period; investigate more distal disorders (e.g., small bowel dysmotility or other pathologies).
  - Activity relieved: increase in gastric activity is associated with reduction in symptoms, suggesting transit or accommodation disorder.
2. Normal spectral analysis and symptoms independent of the gastric amplitude:
  - Continuous profile: symptoms constant/do not correlate with gastric amplitude; consider therapy per epigastric pain syndrome (2).
  - Meal induced: symptoms worse after a meal and not meeting criteria of amplitude-dependent profiles.
  - Meal relieved: symptoms improving after a meal and not meeting criteria of amplitude-dependent profiles.

### Management framework

**Figure 1.**
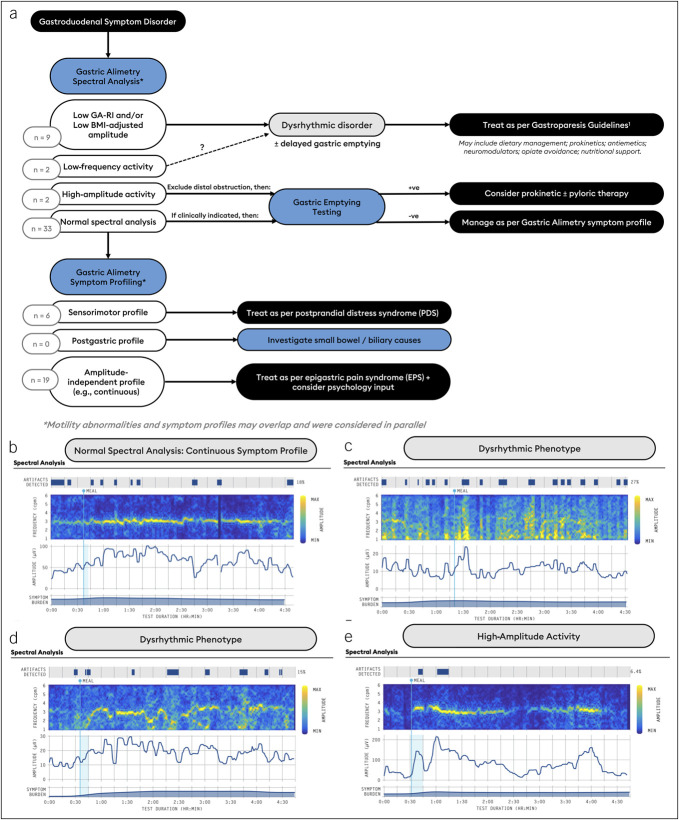
(**a**) Flow diagram developed for the application of Gastric Alimetry in the management of gastroduodenal disorders. Patients may have more than 1 phenotype. (**b–d**) Examples of Gastric Alimetry spectral data, amplitude plots, and symptom burden graphs from the study cohort. (**b**) Normal spectral analysis; continuous symptom profile: principal gastric frequency 3.01 cpm (range 2.65–3.35); Gastric Alimetry-Rhythm Index (GA-RI) 0.88 (range ≥0.25); body mass index–adjusted amplitude 61.3 ± 17.8 μV (range 22–70 μV). (**c**) Dysrhythmic profile (GA-RI 0.12; 21.6 μV). (**d**) Dysrhythmic neuromuscular profile with a sensorimotor symptom profile (GA-RI 0.12; 21.6 μV). (**e**) High amplitude activity (average 72.1 μV).

A framework was iteratively developed on the basis of clinical experience accrued through the presented series (Figure 1a). The management framework is proposed to integrate Gastric Alimetry, GET, and existing clinical guidelines. The model was applied pragmatically with reference to individual patient histories, clinical workup, and comorbidities, while accepting that gastroduodenal disorders and phenotypes may overlap (2).

### Health care utilization analysis

A health care utilization and health economic analysis was also conducted in the major participating center, where follow-up data were available. The average health care utilization costs per patient were calculated before vs after Gastric Alimetry testing. Follow-up was restricted to a year before and after Gastric Alimetry testing. Pre- and post- comparisons were made using paired nonparametric Wilcoxin tests.

## RESULTS

A total of 50 patients were evaluated (Wellington/North Shore Hospitals, New Zealand), 45 were women (90%); median age 30 years (interquartile range [IQR] 22–46). The median follow-up of patients was 12.6 months (IQR 10.8–17.5). Predominant presenting symptoms were nausea/vomiting (72%), upper abdominal pain (8%), other functional gastrointestinal symptoms (e.g., bloating, fullness, heartburn; 20%). No adverse events associated with Gastric Alimetry testing were observed.

### Gastric Alimetry testing

Figure 1b shows representative examples of phenotypes encountered in the cohort. Nine of 50 (18%) had a dysrhythmic disorder, 2/50 (4%) had a high sustained body mass index–adjusted amplitude, and 33 (66%) had normal spectral analysis. Of those with a normal spectral analysis, 9 had symptoms related to the gastric amplitude: 6 (18%) sensorimotor pattern, 3 (9%) activity-relieved pattern; while 19 (58%) had symptoms independent of gastric amplitude (10 [53%] continuous pattern, 7 [37%] meal-induced pattern, and 2 [11%] meal-relieved pattern). The median total symptom burden score was 24.6 (IQR 14.9–31.5).

### Gastric emptying

Most of the patients had a past GET (n = 36; 72%), of which 19 (53%) had delayed emptying, 3 (8.3%) had rapid emptying, and 3 (8.3%) were indeterminate. Ten had a gastric emptying study initiated after Gastric Alimetry testing (of which 8 were repeat tests).

### Changes in clinical management

**Figure 2.**
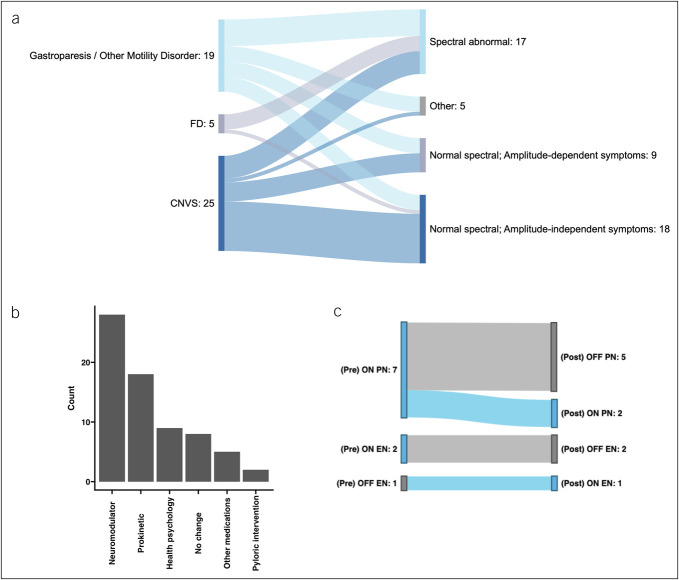
(**a**) Sankey diagram showing the changes in diagnoses that occurred between gastroparesis/other motility disorder and Rome-IV gastroduodenal disorders vs post–Gastric Alimetry diagnoses (right column). (**b**) Significant changes in management that were guided by Gastric Alimetry test data, as per the workflow in Figure 1a. (**c**) Significant changes in invasive (parenteral [PN] or enteral nutrition [EN]) that were achieved because of the changes instituted in Figure 2a, b in tandem with integrated clinical care. CNVS, chronic nausea and vomiting syndrome; FD, functional dyspepsia.

Primary diagnoses before Gastric Alimetry evaluation were gastroparesis/other gut motility disorder (20/50; 40%) or Rome-IV gastroduodenal disorder, 24 (48%) with CNVS, and 6 (12%) with FD. Diagnostic outcomes of the Gastric Alimetry tests are summarized in Table 1 at the patient level and presented in Figure 2a, with impact on diagnosis and management presented in Figure 2b, c. Overall, the test aided a management decision in 42/50 patients (84%). Only 5 (10%) remained unclassified by the Gastric Alimetry phenotyping system, 4 of whom had gastroparesis and 1 had CNVS. Of the 19 with normal spectral analysis, and symptoms independent of gastric amplitude, 5 (26%) had delayed gastric emptying while the remaining 14 (74%) had CNVS or FD (Table 1 and Figure 2a). Medications were de-escalated in 10 (20%). Therapeutic changes favored prokinetics in 36%, neuromodulators in 56%, with 2 patients receiving pyloric intervention, and management focused predominantly on other medications in 8% (Figure 2b).

**Table 1.** GA series

Site	Demographics (age/sex)	Time-of-test dominant symptoms	Total symptom burden index	Diagnosis	GA symptom profile	GA spectral phenotype	GA overall phenotype	Gastric emptying result	Change in investigation	Change in management
Site 1	19 F/20.6	Nausea, bloating	11.4	CNVS	Meal induced	Low rhythm stability	Low rhythm stability	Indeterminant	—	Started prokinetics, started neuromodulators
Site 1	38 F/34.1	Excessive fullness, bloating, nausea, upper gut pain	25.6	Gastroparesis	Meal induced	High frequency	High frequency	Delayed	OGD	Started migraine medications, stopped prokinetics
Site 1	36 F/32.3	Nausea, excessive fullness	23.2	Gastroparesis	Meal induced	Normal spectral metrics	Meal induced	Delayed	—	Increased prokinetics, started neuromodulators
Site 1	28 F/23.7	Excessive fullness, bloating, nausea, stomach burn	27.1	CNVS	Sensorimotor	Normal spectral metrics	Sensorimotor	Normal	—	Increased neuromodulators
Site 1	46 F/29.9	Nausea, bloating, excessive fullness	24.8	Gastroparesis	Meal induced	Low rhythm stability	Low rhythm stability	Delayed	—	Started 5-HT4 agonist
Site 1	30 F/19.2	Nausea, upper gut pain, bloating, excessive fullness	41.6	Gastroparesis	Sensorimotor	Normal spectral metrics	Sensorimotor	Delayed	—	Increased neuromodulators, started migraine medications, stop prokinetics, health psychology input
Site 1	36 F/36.7	Nausea, bloating, excessive fullness, upper gut pain	30.4	Gastroparesis	Continuous	Normal spectral metrics	Continuous	Delayed	—	Increased prokinetics, started neuromodulators
Site 1	22 F/28.8	Nausea, upper gut pain, bloating, excessive fullness	28	CNVS	Meal induced	Low rhythm stability	Low rhythm stability	Indeterminant	GET repeated	Started prokinetics, started 5-HT4 agonists, started neuromodulators, PN stopped
Site 1	64 M/18.5	Excessive fullness, nausea, upper gut pain, bloating	23.9	CNVS	Meal induced	Low amplitude	Low amplitude	Rapid	—	No change
Site 1	22 F/28.1	Nausea, bloating	15	CNVS	Continuous	Normal spectral metrics	Continuous	Rapid	—	Increased neuromodulators
Site 1	30 M/23.5	Nausea, bloating, upper gut pain, excessive fullness	27	FD	Meal induced	Low amplitude	Low amplitude	Normal	—	Started PPI, started muscle relaxant
Site 1	19 F/19.4	Bloating, upper gut pain, excessive fullness, nausea	26.2	CNVS	Meal induced	Low amplitude	Low amplitude	Rapid	—	Start 5-HT4 agonist
Site 1	18 F/21.5	Nausea, bloating, upper gut pain, heartburn, excessive fullness	38.6	Other motility disorder	Sensorimotor	Normal spectral metrics	Sensorimotor	Normal	GET repeated	Started neuromodulator, started migraine meds, health psychology input, PN stopped
Site 1	30 F/18.6	Nausea, bloating, excessive fullness	27.8	Gastroparesis	Sensorimotor	High amplitude	High amplitude	Delayed	GET repeated	Increased neuromodulator, started migraine meds, health psychology input
Site 1	30 F/15.6	Nausea, excessive fullness, stomach burn	39.1	FD	Sensorimotor	Low frequency	Low frequency	N/A	GET repeated	Started prokinetics, started PPI, started neuromodulator, weaned opiates
Site 1	22 F/20.2	Bloating, upper gut pain	15.3	CNVS	Continuous	Normal spectral metrics	Continuous	Normal	—	Health psychology input
Site 1	51 F/23.5	Nausea, bloating, upper gut pain, excessive fullness	28.6	Other motility disorder	Meal induced	Low rhythm stability	Low rhythm stability	Delayed	—	Increased prokinetics, increased neuromodulator
Site 1	60 F/31.6	Excessive fullness	5.7	Gastroparesis	Other	Normal spectral metrics	Other	Delayed	OGD	Pyloric botox
Site 1	30 F/32.7	Nausea, heartburn, bloating, excessive fullness	25.4	Gastroparesis	Other	Low frequency	Low frequency	Delayed	—	Started prokinetics, started neuromodulators, started 5-HT4 agonist
Site 1	45 F/15.0	Bloating	6.6	Gastroparesis	Other	Normal spectral metrics	Other	Delayed	—	Stopped a diabetic medication
Site 1	26 F/17.2	Nausea, excessive fullness	18.9	Gastroparesis	Sensorimotor	Normal spectral metrics	Sensorimotor	Delayed	—	Increased neuromodulator, stopped 5-HT4 agonist, started antiemetics, PN stopped
Site 1	46 F/20.8	Excessive fullness, heartburn, stomach burn, bloating	34.8	FD	Meal induced	Normal spectral metrics	Meal induced	N/A	—	Started neuromodulator
Site 1	32 F/21.9	Excessive fullness, bloating, nausea	11.9	CNVS	Meal induced	Normal spectral metrics	Meal induced	N/A	GET repeated, OGD	Increased neuromodulators, started prokinetics, stopped opiates
Site 1	32 F/20.8	Nausea, upper gut pain	4.1	CNVS	Sensorimotor	Normal spectral metrics	Sensorimotor	Indeterminant	GET repeated, OGD	No change
Site 1	21 F/23.0	Bloating, nausea, stomach burn, excessive fullness, heartburn, upper gut pain	24.3	CNVS	Activity relieved	Normal spectral metrics	Activity relieved	Normal	—	Started prokinetics
Site 1	17 F/19.4	Bloating, excessive fullness, nausea	14.8	CNVS	Activity relieved	Normal spectral metrics	Activity relieved	N/A	GET, OGD	No change
Site 1	70 M/23.4	Bloating, excessive fullness	17.6	CNVS	Activity relieved	Normal spectral metrics	Activity relieved	Normal	—	No change
Site 1	23 M/21.4	Nausea, excessive fullness	19.4	CNVS	Meal induced	Normal spectral metrics	Meal induced	N/A	OGD	Started neuromodulators, start 5-HT4 agonist
Site 1	46 F/23.4	Excessive fullness, nausea, upper gut pain	31.6	Gastroparesis	Meal induced	Normal spectral metrics	Meal induced	Delayed	GET repeated	Increased prokinetics, started neuromodulator, start 5-HT4 agonist, PN stopped
Site 1	51 F/22.7	Bloating, stomach burn	6.8	FD	Other	High frequency	High frequency	Normal	—	Start PPI, start prokinetics, start neuromodulator
Site 1	63 F/19.6	Bloating, upper gut pain	5.7	Gastroparesis	Other	Low rhythm stability	Low rhythm stability	Delayed	Antroduodenal manometry	Other (increase octreotide), health psychology input
Site 1	19 F/25.0	Bloating, nausea, upper gut pain	23.8	CNVS	Continuous	Normal spectral metrics	Continuous	N/A	—	Start neuromodulators
Site 1	25 F/18.9	Excessive fullness, bloating	7.1	Gastroparesis	Other	Normal spectral metrics	Other	Delayed	—	No change
Site 1	30 F/29.5	Nausea, bloating, upper gut pain, excessive fullness	29.5	CNVS	Meal induced	Normal spectral metrics	Meal induced	Normal	—	Increase PPI, increase neuromodulators
Site 2	26 F/22.0	Nausea, excessive fullness, upper gut pain, bloating	39.6	Gastroparesis	Continuous	Normal spectral metrics	Continuous	Normal	—	Started H1 receptor antagonist, started prokinetics, PN stopped
Site 2	50 F/20.7	Nausea, upper gut pain, heartburn, stomach burn, bloating, excessive fullness	59.1	CNVS	Continuous	Normal spectral metrics	Continuous	Normal	—	Increased neuromodulator, stopped 5-HT3 antagonist
Site 2	23 F/31.2	Nausea, bloating, heartburn	15.1	Gastroparesis	Other	Normal spectral metrics	Other	Delayed	—	No change
Site 2	20 F/25.1	Stomach burn, nausea, excessive fullness	18.5	CNVS	Meal relieved	Normal spectral metrics	Meal relieved	Normal	GET repeated, MRE	Increased neuromodulator, stopped H2 receptor antagonist
Site 2	44 F/30.1	Heartburn, stomach burn, upper gut pain, nausea, bloating, excessive fullness	31	Gastroparesis	Meal induced	High amplitude	High amplitude	Delayed	GET	No change
Site 2	18 F/20.4	Stomach burn, heartburn, excessive fullness, upper gut pain, nausea, bloating	41.1	FD	Sensorimotor	High frequency	High frequency	N/A	—	Increased PPI
Site 2	56 F/18.3	Bloating, excessive fullness	13.5	FD	Meal induced	Normal spectral metrics	Meal induced	N/A	—	Started neuromodulator, other (stopped pancreatic enzymes), health psychology input, immunology input, dietitian
Site 2	34 F/29.9	Excessive fullness, bloating, nausea	13.3	CNVS	Meal relieved	Normal spectral metrics	Meal relieved	N/A	—	No change
Site 2	53 F/39.7	Bloating, excessive fullness, upper gut pain, nausea, stomach burn	36.3	CNVS	Continuous	High frequency	High frequency	N/A	—	Started prokinetics
Site 2	29 F/36.8	Upper gut pain, heartburn, stomach burn, excessive fullness	36.7	CNVS	Meal induced	Low rhythm stability	Low rhythm stability	N/A	—	Started neuromodulator
Site 2	58 M/27.1	Bloating, excessive fullness, nausea	7.4	CNVS	Continuous	Normal spectral metrics	Continuous	Normal	—	Increased prokinetics, increased neuromodulator, increased PPI
Site 2	63 F/24.5	Upper gut pain	1.6	CNVS	Other	Normal spectral metrics	Other	N/A	—	Increased skeletal muscle relaxants, health psychology input, diaphragmatic breathing
Site 2	19 M/20.4	Nausea, bloating, heartburn	14.7	CNVS	Continuous	Normal spectral metrics	Continuous	N/A	—	Increased neuromodulator, increased prokinetics, started antidepressant, health psychology and psychiatry input
Site 2	25 F/17.6	Excessive fullness, nausea, heartburn, bloating, upper gut pain	25.6	Gastroparesis	Continuous	Normal spectral metrics	Continuous	Delayed	—	Increased neuromodulator, increased prokinetics
Site 2	25 F/16.6	Nausea, bloating, upper gut pain, heartburn, stomach burn, excessive fullness	51.6	CNVS	Continuous	Normal spectral metrics	Continuous	N/A	—	Increased neuromodulator, dietitian, health psychology and psychiatry involvement
Site 2	18 F/19.9	Bloating, nausea, excessive fullness, upper gut pain	33.9	Gastroparesis	Sensorimotor	Normal spectral metrics	Sensorimotor	Delayed	—	Pyloric intervention recommended

5-HT, 5-hydroxytryptamine; CNVS, chronic nausea and vomiting syndrome; FD, functional dyspepsia; GA, Gastric Alimetry; GET, gastric emptying test; MRE, magnetic resonance enterography; N/A, not applicable; OGD, Oesophago-Gastro-Duodenoscopy; PN, parenteral nutrition; PPI, proton pump inhibitor.

Changes in invasive nutritional support occurred in 9/50 cases (18%) (Figure 2c). Of 7 patients on parenteral nutrition before evaluation, 5 were successfully weaned, together with 3 patients on enteral feeding, after management changes facilitated tolerance of diet. Conversely, nasojejunal feeding was initiated in 1 patient with a neuromuscular disorder who failed to respond to medical management.

### Health care utilization

**Figure 3.**
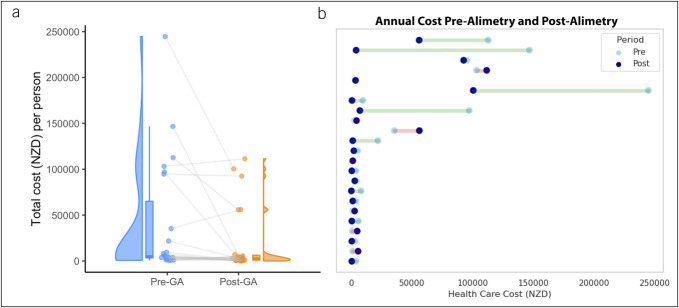
(**a**) Pre- and post-Gastric Alimetry (GA) testing patient-level health care utilization cost savings. Total costs incurred per person in the year before and after GA testing per person (*P* = 0.037). (**b**) Dumbbell plot of patient-level cost differences pre-GA and post-GA testing. Costs in New Zealand dollars. Each line represents an individual participant with follow-up data available at the major participating center.

Follow-up data were available for 23/37 of the patients in the major participating center, allowing a health care utilization analysis. The remaining patients without follow-up data were referred from external centers and/or had a substantial portion of their care undertaken in another country. The median follow-up was 336 days (IQR 283–435) after Gastric Alimetry testing. The number of investigations, emergency department visits, clinic visits, and inpatient stays reduced after Gastric Alimetry testing, compared with pretesting health care utilization (Table 2). The average health care utilization cost was $39,724 ± 63,566 (median $5,095, IQR $3,421.6–$65,082) in the year before Gastric Alimetry compared with an average cost of $19,937 ± 35,895 (median $2,844, IQR $1,043–$6,244) in the year after (*P* = 0.037), with a significant contribution from de-escalation of care including invasive nutrition. This represents a 50% average cost reduction per person translating to a per-person cost saving of $19,787. Differences between pre–Gastric Alimetry testing and post–Gastric Alimetry testing at the patient level are visualized in Figure 3.

**Table 2.** Health care utilization before and after Gastric Alimetry testing

	Before Gastric Alimetry testing (frequency per year per patient)			After Gastric Alimetry testing (frequency per year per patient)		
	Total n	n per year	Cost (NZD)	Total n	n per year	Cost (NZD)
Diagnostic tests						
Abdominal CT scan	28	0.28	244.85	6	0.09	76.04
Abdominal MRI scan	6	0.06	103.31	2	0.03	48.88
Abdominal x-ray	145	1.61	252.47	22	0.32	50.14
Barium study	4	0.05	29.95	0	0	0
Brain CT scan	5	0.06	53.71	0	0	0
Brain MRI scan	4	0.03	55.13	0	0	0
CT NOS	4	0.04	32.38	2	0.03	25.35
MRI NOS	1	0.02	35.13	1	0.01	24.44
Ultrasound	25	0.27	80.51	6	0.09	26
Colonic transit study	1	0.01	12.89	0	0	0
Gastric emptying study (scintigraphy)	18	0.16	425.52	6	0.09	230.43
Gastroscopy	55	0.58	802.54	35	0.78	1,083.31
Colonoscopy	11	0.16	322.05	1	0.01	30.08
Endoscopy NOS	4	0.03	46.91	1	0.01	20.06
ED visits						
ED stay with transfer to inpatient	76	0.78	1,017.39	17	0.38	499.28
ED visit: duration 0–2 h	1	0.02	6.67	1	0.08	33.33
ED visit: duration 2–23 h	63	0.82	618.42	11	0.43	326.09
Interventions						
Feeding tube	45	0.44	44.02	19	0.28	27.54
TPN days	3,431	37.11	8,569.25	904	17.16	3,963.12
Clinic visits						
GI clinical appointment (initial)	29	0.3	204.52	3	0.04	29.38
GI clinical appt (follow-up)	167	1.64	632.11	79	1.21	468.25
Medical inpatient (day stay)	25	0.24	145.59	15	0.22	130.43
Medical inpatient (overnight stay)	94	0.9	1,077.37	26	0.51	617.39
No follow-up	97	0.98	0	73	1.33	0

CT, computed tomography; ED, emergency department; GI, gastrointestinal; MRI, magnetic resonance imaging; NOS, not otherwise specified; TPN, total parenteral nutrition.

## DISCUSSION

These data present the first real-world report of the introduction of Gastric Alimetry into a clinical care pathway. The results showed that most (84%) of the patients achieved a positive impact from the new test, with the most consequential changes being a switch from a gastroparesis or other motility disorder to normal gastric rhythm group (63% of patients). These changes were justified because GET is now established to be insensitive and nonspecific for neuromuscular abnormalities, whereas Gastric Alimetry is targeted toward neuromuscular profiling, regardless of GET status (3,5,13). It should also be noted that the test was only an aid to decision-making, and participating clinicians also considered other patient data at follow-up, which likely contributed to the changes observed in clinical care.

These results demonstrate the potential for an alternative paradigm in managing gastroduodenal symptoms, arising from incorporating the Gastric Alimetry test. In particular, the framework reduced reliance on symptom criteria and GET, which present overlapping profiles (3), and instead proposed phenotyping patients based on specific mechanisms when possible (5,6,13). The phenotypes applied here cover several known gastroduodenal pathologies, including neuromuscular disorders (including interstitial cell of Cajal pathologies), visceral hypersensitivity, disorders of gut-brain interaction, and gastric outlet resistance (10). While these disorders are well established, they have previously been challenging to separate and apply at the individual patient level. Mechanistic phenotyping then facilitated personalized therapy.

It is noted that the proposed management framework attempts to combine existing guidelines and current practice when appropriate to adapt best practice recommendations. For example, neuromodulators are not recommended for gastroparesis management by the American College of Gastroenterologists (1). However, the Rome foundation recommendations for disorders of gut-brain interactions and clinical practice commonly include the use of neuromodulators in these overlapping disorders (14). Further prospective studies using Gastric Alimetry phenotyping are desirable to attempt selection for patients most likely to benefit from these therapies. Similarly, we also envision the newly objective and reproducible biomarkers of gastric function to be useful to direct tailored interventions, such as pyloric intervention in cohorts where outlet resistance may be contributory (10,13,15). For example, patients with normal or high-amplitude gastric electrophysiology in the presence of delayed gastric emptying could be appropriate candidates for pyloric approaches (13,16). Randomized trials and large-scale databases are anticipated to further investigate these hypotheses.

The changes in care pathways described in this study may have substantial implications for outcomes, costs of care, and quality of life in gastroduodenal disorders. The introduction of Gastric Alimetry into the clinical care pathway for chronic gastroduodenal disorders was associated with reduced health care utilization and health economic advantages to the benefit of both patients and the health care system. Data indicated that the reduction in costs occurred across diagnostic testing, emergency department and clinic visits, and frequency of interventions. In addition, one key driver of patient benefit and decreased health care costs was reduced invasive nutrition including parenteral nutrition. However, it is also emphasized that this study mainly focused on diagnostic and management pathway changes, and prospective studies evaluating additional patient outcome data are now justified (12,17).

This series reflects a first overview of the impact of integration of Gastric Alimetry into routine gastroenterology practice, synthesized within the first 2 tertiary referral services to apply the test internationally. Gastric Alimetry is a new test, and we anticipate that the new model will be refined and improved as use of the device expands. Ongoing work, in consultation with experts in the field, is also occurring to develop a consensus-driven phenotyping and classification set to enable standardized application and interpretation of Gastric Alimetry tests in clinical practice (10).

Short-term and long-term improvements in symptoms were beyond the scope of this study, but future prospective trials with comparator groups should focus on validating the proposed framework developed here. In this study, we quantify the decisions made upon addition of the Gastric Alimetry testing and interpretation of arising reports. It is beyond the scope of this study to attribute causality to positive outcomes; and instead, the descriptive changes in diagnosis, interventions, and management should be understood as associations. In addition, a potential limitation is that the use of a before vs after analysis for health care utilization could be subject to bias, as it might be expected that test usage may drop over time for patients being subject to a diagnostic workup. However, in practice, evidence does suggest that high health care utility burdens usually do continue at high sustained levels in chronic gastroduodenal disorders (18,19).

In summary, this study represents the first real-world clinical series of patients with chronic gastroduodenal investigated with Gastric Alimetry. Data show that this test holds significant clinical potential, with positive changes observed in management occurring in the high majority of cases, reductions in invasive nutrition, and substantially reduced health care utilization. Further studies should now focus on prospective assessments of the individual phenotype-based management introduced here.

## CONFLICTS OF INTEREST

**Guarantor of the article:** Chris Varghese, MBChB.

**Specific author contributions:** All authors were involved in the conception, data collection, analysis, and writing of the article.

**Financial support:** This project was supported by the Health Research Council of New Zealand.

**Potential competing interests:** None to report.

**Data availability statement:** Data used for analysis will be made available on reasonable request, conditional on ethical approvals.

**Study Highlights**

**WHAT IS KNOWN**

✓ Diagnostic classifications for chronic gastroduodenal symptoms are overlapping, limiting targeted treatments.

**WHAT IS NEW HERE**

✓ Gastric Alimetry aided the diagnosis and management of patients with chronic gastroduodenal symptoms, reducing health care utilization.

## Supplementary Material
